# Yes, I Can: The Interplay of Need for Cognition and Task Confidence in Cognitive Task Performance

**DOI:** 10.3390/jintelligence12120128

**Published:** 2024-12-15

**Authors:** Monika Fleischhauer, Felix M. Schweitzer, Sören Enge

**Affiliations:** Department of Psychology, MSB Medical School Berlin, D-14197 Berlin, Germany; felix.m-schweitzer@dozent.medicalschool-berlin.de (F.M.S.); soeren.enge@medicalschool-berlin.de (S.E.)

**Keywords:** Need for Cognition, task performance, task-specific self-efficacy, performance visibility, moderated mediation

## Abstract

Need for Cognition (NFC) refers to the enjoyment of and the search for intellectual challenges. Although numerous studies suggest associations between NFC and cognitive performance, the processes and factors that may mediate the relationship are not yet well understood. Based on the literature suggesting that self-efficacy (SE) expectancies mediate the relationship between NFC and cognitive performance, we sought to investigate this relationship systematically under controlled laboratory conditions. Additionally, we were interested in whether the visibility of the test subject’s performance to others (i.e., the experimenter) would influence these correlations. After an online questionnaire assessing NFC, 204 participants completed a set of highly demanding intelligence tasks in the laboratory. Following the sample tasks and prior to working through the task battery, task-specific SE about solving the tasks was assessed. To examine the role of visibility, participants either worked alone or were observed by the investigator while completing the tasks. We found a moderate positive association between NFC and task-specific SE, as well as a significant small positive association between NFC and task performance. Further analyses indicated that the relationship between NFC and task performance is fully mediated by task-specific SE, without any moderation effects related to the visibility of one’s own task performance to others. Our study suggests that the relationship between NFC and cognitive performance is also due to the positive influence of NFC on task-specific SE, which in turn influences cognitive performance.

## 1. Introduction

Need for cognition (NFC), defined as an individual’s tendency to seek out and enjoy cognitive endeavors (Cacioppo et al. 1984), has been shown in numerous studies to be associated with academic success (e.g., Liu and Nesbit 2024; Strobel et al. 2024), psychometric intelligence (e.g., Fleischhauer et al. 2010; Hill et al. 2013), and complex skill acquisition (Day et al. 2007). Although the processes underlying these associations remain largely unexplored, results from previous studies suggest that self-efficacy (SE) may mediate the relationship between NFC and cognitive performance (e.g., Elias and Loomis 2002; Naderi et al. 2018). To date, however, there has been no study investigating the interplay between NFC and the SE in solving specific cognitively demanding tasks. The present experimental study aims to address this gap. Specifically, we seek to determine whether individuals with higher NFC demonstrate increased confidence in their ability to solve cognitively demanding tasks and whether this task-specific SE influences their task performance by acting as a mediator in the relationship between NFC and performance. Additionally, we will examine whether the visibility of the task process to others affects the performance levels of individuals with varying levels of NFC and SE. Through this analysis, we aspire to gain a deeper understanding of the cognitive and motivational mechanisms that shape the relationship between NFC and cognitive performance.

### 1.1. Background

Need for Cognition (NFC) is defined as the “tendency to engage in and enjoy effortful cognitive processing” (Cacioppo and Petty 1982, p. 116). Individuals with lower NFC, referred to as “cognitive misers” (Cacioppo et al. 1996, p. 197), tend to minimize cognitive effort in information processing. They are more likely to employ cognitive shortcuts to quickly understand information, for example, by trusting the status of an information source rather than carefully scrutinizing the information (Priester and Petty 1995). In contrast, individuals with higher levels in NFC, also called “chronic cognizers” (Cacioppo et al. 1996, p. 197), seek out complex problems, enjoy analyzing information, and prefer tasks that require elaborative thinking. Thus, these individuals are more likely to engage in effortful cognitive endeavors and problem-solving activities, and they typically find intellectual challenges enjoyable (for an overview, see Cacioppo et al. 1996; Petty et al. 2009).

It is evident that the main characteristic of NFC—the active pursuit and enjoyment of challenging intellectual tasks—may be associated with advantages in knowledge acquisition and cognitive development. Numerous studies show that NFC is positively associated with elaborative information processing and cognitive performance measures. For instance, individuals with higher NFC not only search for information more intensively (e.g., Mokhtari et al. 2013; Verplanken et al. 1992), but also comprehend information better and more quickly (Jebb et al. 2016) than their low NFC counterparts. Moreover, they are less susceptible to cognitive biases (Palmer and Feldman 2005) and their decision-making tends to be more accurate (e.g., Carnevale et al. 2011; Levin et al. 2000) and is based more on relevant information (Dickhäuser et al. 2009). Furthermore, NFC shows small to moderate positive associations with tests of fluid intelligence (e.g., Fleischhauer et al. 2010), crystallized knowledge (Tidwell et al. 2000; Wood and Bandura 1989), and academic performance (Grass et al. 2017; Strobel et al. 2024; for a meta-analysis, see Liu and Nesbit 2024). These associations appear to be somewhat higher for the fluid aspects of intelligence than for crystallized knowledge (Fleischhauer et al. 2010) and academic performance (Liu and Nesbit 2024), which would also be assumed by the Openness–Fluid–Crystallized-Intelligence (OFCI) model of Ziegler et al. (2012) assuming a more direct relationship between the NFC-related factor of openness to experience (O) and fluid intelligence than between O and crystallized intelligence.

There is growing interest in the processes and factors that may mediate the relationship between NFC and cognitive performance. For example, Bertrams and Dickhäuser (2009) found that self-control—defined as the ability to prioritize long-term advantageous global goals over short-term situational advantages—was not only significantly associated with NFC, but also mediated the relationship between NFC and the school grades of the participants. However, in their attempt to clarify the relationship between NFC and central executive functions (inhibitory control, shifting, updating), Gärtner et al. (2021) found no significant relationship between NFC and any of these functions in their study, making the role of pure executive control factors as mediators less likely. The authors conclude that NFC may be characterized rather by the way they allocate their cognitive resources than by a better executive functioning. Studies using electroencephalographic measures show that individuals with higher NFC invest more mental effort compared to those with lower NFC (Enge et al. 2008; Mussel et al. 2016). Moreover, there is evidence that they are more motivated to avoid failure (Steinhart and Wyer 2009) when confronted with higher cognitive demands.

Another factor that may explain the positive association between NFC and academic performance, intelligence, and decisional processes, is self-concept. Higher levels of self-esteem and self-confidence are considered key factors in academic achievement (Golden 2003). Moreover, a meta-analysis by Wu et al. (2021) demonstrated that academic self-concept and academic achievement are reciprocally related. That is, the academic self-concept is not only shaped by experiences of academic success, but also influences future academic performance. According to Bless et al. (1994), individuals with higher NFC tend to have a more favorable assessment of their cognitive abilities. This is supported by Grass (2018), who showed that NFC had small to moderate positive correlations with self-estimated intelligence, with the strongest correlation observed in the domain of logical reasoning. Similar results were reported by Strobel et al. (2024), who found a strong positive correlation between NFC and the overall ability self-concept of pupils (*r* = 0.50).

A conceptually similar motivational construct, which also serves as a coping resource in dealing with challenging situations (see Schwarzer and Jerusalem 2002; Vollrath 2001) and which is positively related to performance in cognitive tasks (Bouffard-Bouchard 1990), is self-efficacy (SE). SE is defined as the “beliefs in one’s capabilities to mobilize the motivation, cognitive resources, and courses of action needed to meet given situational demands” (Wood and Bandura 1989, p. 408). Individuals with high SE tend to pursue more ambitious goals and are more willing to persevere and exert effort to successfully complete tasks in the face of obstacles (e.g., Bandura 1977). Theoretically, it can be argued that NFC and SE are closely linked, as individuals with high NFC are more likely to be motivated to solve complex problems and actively engage with difficult material, which in turn could continuously strengthen their belief in their own ability to overcome these challenges and thus increase their SE. Studies indeed showed positive associations between NFC and SE (e.g., Elias and Loomis 2002; Naderi et al. 2018; Pillai et al. 2011; Tan et al. 2020). Furthermore, SE has been demonstrated to mediate the relationship between NFC and various performance outcomes, including academic performance (Elias and Loomis 2002), complex skill acquisition in a video game (Day et al. 2007), and academic burnout (Naderi et al. 2018). Thus, SE appears to be a relevant motivational factor that elucidates how NFC influences cognitive performance by fostering confidence in one’s abilities and motivation to tackle challenging tasks, and that may help to understand the frequently observed correlations between NFC and performance outcomes. Prior studies that investigated the interplay between NFC and SE in explaining cognitive performance mainly focused on general SE (Naderi et al. 2018; Pillai et al. 2011), referring to an individual’s belief in their overall competence across various situations (e.g., Chen et al. 2004). However, to our knowledge, there has been no study investigating the interplay between NFC and self-efficacy in relation to a specific cognitively demanding task (task-specific SE). This was the aim of the present study.

### 1.2. Hypotheses and Research Question

Given the aforementioned empirical evidence on the relationship of NFC, SE, and cognitive performance, we posed the following hypotheses:

**Hypothesis** **1.** *Individuals with higher NFC levels exhibit higher levels of task-specific SE.*

**Hypothesis** **2.** *Individuals with higher NFC levels perform better in cognitively demanding tasks.*

**Hypothesis** **3.** *Task-specific SE acts as mediator in the relationship between NFC and task performance.*

Besides the question whether task-specific SE acts as a mediator in the relationship between NFC and task performance, we were further interested in the question whether the visibility of the task process to others affects the relationship between NFC and task performance. We implemented two task settings: in the working-alone setting, participants finished the cognitive tasks alone, while in the observation setting, they were observed by the experimenter. Numerous studies indicate that the pressure to perform in front of others can induce nervousness or anxiety. This emotional response may impair performance, especially when tasks are perceived as challenging or novel (e.g., Rezaei et al. 2017). Since NFC is defined as an intrinsic motivation to think, and individuals with high NFC possess a strong interest in and enjoyment of thinking and learning (Cacioppo et al. 1996), one might assume that they feel less pressured by the observation of others compared to individuals with low NFC. Consequently, the performance differences in the presence of others may be even more pronounced than in the working-alone setting.

However, it is equally conceivable that the presence of observers increases motivation to solve cognitive tasks as individuals intend to avoid failure and present a positive public image and gain a better social standing (Huang and Yu 2018). Regarding NFC, Steinhart and Wyer (2009) were able to demonstrate that the motivation to avoid failure as a negative outcome increased in individuals with higher NFC when they anticipated a difficult task, but not in those with lower NFC scores. The authors, however, suggested that these results could be interpreted as indicating that the motivation of individuals with higher NFC may manifest in their desire to avoid self-perceptions of incompetence. As a positive self-perception should be relevant in both the working-alone and the observation setting, task-performance of individuals high in NFC should be independent of these settings. Moreover, studies on Need for Cognition (NFC) and effort discounting (Westbrook et al. 2013; Zerna et al. 2023) suggest that individuals with higher NFC scores tend to engage in more challenging tasks and attribute a greater intrinsic value to these tasks compared to individuals with lower NFC scores. Additionally, Thompson et al. (1993) showed that external rewards tended to hinder task performance in high NFC individuals, whereas the opposite was true for low NFC individuals, suggesting that low NFC individuals may be more likely to benefit from the observation setting.

Since the conceptualization of NFC and the empirical findings presented here would allow for divergent hypotheses, we take an exploratively approach and pose the following research question: Does the relationship between NFC and task performance depend on the visibility to others?

## 2. Materials and Methods

### 2.1. Procedure

Data were collected as part of a larger research project. Participants were recruited via SONA, an online research recruitment application utilized by the university, as well as through personal networks of the experimenters. Data collection was divided into two parts. In a first part, participants completed an online questionnaire provided through the Tivian software (v21.1-24.2). This questionnaire took about 40 min to complete. At the beginning of the survey, participants provided informed consent and created an individual participant code to allow us to match the data from the first and the second part of the study, while ensuring anonymity. Participants then completed several questionnaires measuring personality traits, including NFC. Additionally, several excluding criteria (e.g., severe physical, neurological, and mental disorders) were assessed, as this study was part of a more comprehensive research project examining the relationship between stress measures, such as salivary cortisol, and personality.

**Figure 1:**
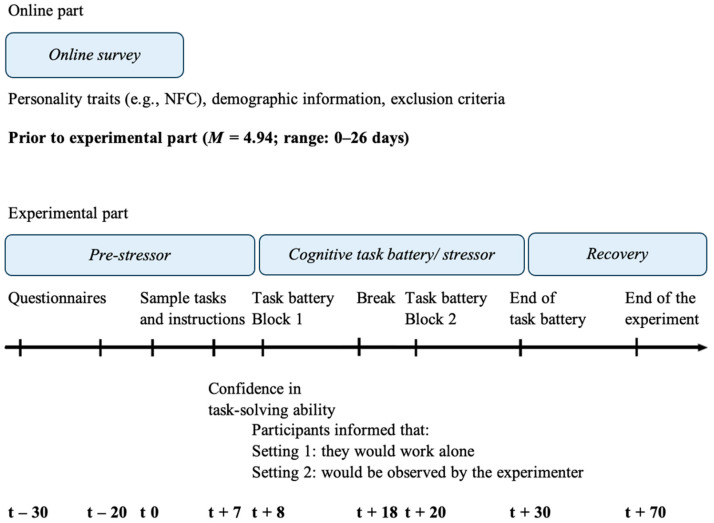
Procedure.

Several days (*M* = 4.94 days; range: 0–26) after completing the online survey, the second part of the study took place in the university labs. The experimental procedure is illustrated in Figure 1. Upon arrival at the laboratory, participants completed several questionnaires, and baseline stress was measured both subjectively (via questionnaire) and objectively (via salivary cortisol). The cognitive task battery, which included four task types (anagram, matrix, cube rotation, and figural analogy tasks), was then explained using sample tasks. The participants were not obliged to solve the sample tasks and, in most cases, did not do so. The purpose of the sample tasks was merely to familiarize the participants with the task material and to give them the opportunity to ask questions. After familiarizing themselves with the sample tasks, participants were asked to assess their confidence in their ability to perform the tasks (task-specific SE). Participants were then asked if they had any questions about the procedure. Questions were answered if necessary, and participants were informed that no further questions would be allowed during the task. They were also instructed to immediately stop working on the tasks when told that time was up. Participants then worked on a series of highly challenging intelligence test tasks for 2 × 10 min with a short break of 2 min in between. In this break, as well as on several other occasions, data on additional subjective stress markers (not relevant for the present study) was collected. After completing the task battery, a recovery phase followed, which was necessary for the salivary cortisol data collection but is not relevant for the present study. In total, the laboratory session lasted approximately 100 min. The study adhered to the Declaration of Helsinki (revised version) and was approved by the ethics committee of the MSB Medical School Berlin (MSB-2022/111).

To investigate whether social contexts like the presence of observers moderate the relationship between NFC and task performance, this study was conducted in two different settings. In setting 1 (working-alone setting) participants were instructed to work alone on the cognitive task battery while the experimenter was outside the lab and only returned to inform them when the time for working on the tasks was up. In setting 2 (observation setting), participants were informed that the experimenter would be present in the room, sitting right next to them, and would note their response behavior to the tasks they were working on at the computer. The answers were recorded by hand on an answer sheet, and the experimenter recorded the time using a stopwatch. This procedure was made plausible by informing participants that they would subsequently receive feedback on their task performance, whereby the results would supposedly have to be documented manually by the experimenter, as they could not be retrieved from the server during the short time of the experimental part. Despite the claim that feedback was obligatory, participants were given the choice to receive it after completing the experiment. We first collected data only using setting 1 from November 2021 until August 2023 and then changed to setting 2, under which data collection is ongoing. It was made certain that none of the participants could take part in both setting 1 and 2.

### 2.2. Sample Characteristics

As described above, this study is part of a larger ongoing research project in which salivary cortisol data are also collected. For this reason, only individuals aged between 18 and 35 years who smoked fewer than 10 cigarettes a day and were not taking cortisone-containing medication were allowed to participate in the study. Additionally, only individuals who had not undergone neurological, psychiatric, psychotherapeutic, or endocrine treatment in the last 12 months and who did not have a mental disorder were included in the sample.

The sample consisted of a total of 204 participants (sex: 75% female, 25% male; gender: 75% female, 1.5% diverse) aged between 18 and 35 years (*M* age = 23.12 years, *SD* = 3.67). Almost all participants (95.6%) reported being currently enrolled at a university, with 96% of these students studying psychology. Of the 204 participants, 99 individuals (69.7% female, 1% divers, *M* age = 24.0, *SD* = 3.9, range = 18–35 years, 96.2% students) completed setting 1, where they worked on the task battery without being observed. The remaining 105 participants (80% female, 1.9% divers, *M* age = 22.3, *SD* = 3.3, range = 18–35 years, 94.9% students) worked on the task battery under the observation of the experimenter. Even though the participants in setting 1 were slightly older and the gender distribution was more balanced than in setting 2, there were no significant differences in our independent variables NFC (*p* = .856) and task-specific SE (*p* = .612).

### 2.3. Measures

NFC was measured using the German version of the NFC scale (Bless et al. 1994), which consists of 16 items (e.g., “I prefer my life to be filled with puzzles that I must solve.”) that must be answered on a 7-point Likert scale ranging from −3 (strongly disagree) to 3 (strongly agree). The NFC score was calculated by forming a mean value. For *N* = 204 valid cases, the scale yielded a Cronbach’s α of .85.

Task-specific SE was assessed after presentation of the sample tasks of each task type and before completing the task battery with the question: “How confident are you that you can handle the (matrix) tasks well?” The four items (one for each task type) had to be answered on a 5-point Likert scale ranging from 1 (not at all) to 5 (extremely). The scale yielded a Cronbach’s α of .77, and the indicator for task-specific SE was calculated by averaging the four items.

The two tasks blocks (each lasting 10 min) featuring cognitively demanding tasks were presented via SoSci-Survey. The tasks included the following: 1. matrix tasks from the Hagen Matrices Test (Heydasch et al. 2013), 2. anagram tasks from Voss (2004)**,** as well as 3. cube rotation tasks and 4. figural analogy tasks, both sourced from the International Cognitive Ability Resource Project (Condon and Revelle 2014; see https://icar-project.com/, accessed on 25 October 2021). The tasks were chosen to provide the participants with a considerable cognitive challenge reflected by the high item difficulties (see below). Moreover, we wanted to target different reasoning aspects (i.e., verbal reasoning with the anagram tasks, non-verbal figural reasoning with the matrix tasks and figural analogy tasks, as well as spatial reasoning with the cube tasks) in order to have a broad representation of reasoning abilities.

In each case, only one correct answer was possible, which could be selected using the cursor or, in the case of the anagram tasks, entered via the keyboard into a designated text field. Participants received each task on a separate page. The order of the tasks was as follows: First, a matrix task was presented, followed by two anagram tasks, a spatial reasoning task, and a figural analogy task. This unit was then repeated, and the order of the presented tasks was the same for all participants. In total, block 1 consisted of 50 cognitively demanding tasks, divided into 10 matrices, 10 spatial reasoning tasks, 10 figural analogy tasks, and 20 anagram tasks. Block 2 included a total of 45 tasks, with 18 being anagram tasks, while the presentation of the other task types was evenly distributed across 9 tasks per type. The number of tasks in block 2 was lower because not all the available sources allowed us to draw the same number of tasks with an item difficulty that was sufficiently high and comparable to that of the other task types. This led to an uneven number of available task units, and we hence allowed block 2 to be slightly shorter, and also to prevent a possible fatigue effect in block 2.

For both the matrices and spatial reasoning tasks, there were six response options, one of which was “I do not know the answer”. Similarly, figural analogy tasks also had six possible responses, with one option stating “The correct answer is missing”. Items from the respective sources were chosen such that mean item difficulty was comparable across the four types, being *M* = 0.35, *SD* = 0.23, range = 0.11–0.85 for the matrices, *M* = 0.34, *SD* = 0.27, range = 0.04–0.86 for the anagrams, *M* = 0.28, *SD* = 0.09, range = 0.12–0.40 for the cube rotation tasks, and *M* = 0.36, *SD* = 0.13, range = 0.12–0.57 for the figural analogy tasks. Overall, the tasks exhibited a lower item difficulty index (*M* = 0.34; *SD* = 0.21, range = 0.04–0.86), indicating that they were challenging for the majority of the population. The average item difficulty across both blocks was kept similar. The internal consistency of the eight indicators (correctly solved tasks from four task types across two blocks) yielded a Cronbach’s α of .60. Task performance as a dependent variable was determined by the number of correctly solved tasks across the four task types and the two task blocks. In the full sample (*N* = 204), participants correctly solved an average of 9.59 out of 95 possible items (*SD* = 4.03, range *=* 0–23). When comparing the two settings, individuals in setting 2 solved slightly more items correctly (*M* = 10.02, *SD* = 4.07, range = 3–22) than those in setting 1 (*M* = 9.14, *SD* = 3.96, range = 0–23). However, this difference was not statistically significant (*t* = −1.559, *p* = .121, 95% CI [−1.988, 0.232]).

### 2.4. Data Analysis

Data were analyzed using IBM SPSS Statistics 25. To examine whether individuals with higher NFC are more confident in their ability to solve cognitively challenging tasks (Hypothesis 1) and whether they are actually able to do so (Hypothesis 2), Pearson correlation analyses were conducted. To analyze the mediation effect of NFC on task performance via task-specific SE (Hypothesis 3), the SPSS macro Process v4.0. (Hayes 2017) was employed. Bootstrapping with 5000 samples was used to reduce the potential risks associated with violations of normal distribution (Field 2013). Effects were deemed significant when the 95% confidence interval did not include zero. Additionally, HC4 heteroscedasticity consistent standard errors (Davidson et al. 1994) were employed. There were no outliers in the data that exceeded three times the interquartile range. Finally, we extended the mediation model by incorporating the moderator variable “setting” to investigate whether the presence vs. absence of the experimenter influenced the effect of NFC on task performance (research question). Prior to calculating the interaction term with the categorical setting variable, NFC and task-specific SE were centered. The dataset generated and analyzed in the current study is available at: https://osf.io/cjq46/ (accessed on 25 November 2024).

## 3. Results

As shown in Table 1, NFC was positively correlated with task-specific SE (*r* = 0.29, *p* < .001). Additionally, there was a small positive association between NFC and task performance (*r* = 0.14, *p* = .049). This suggests that with increasing NFC, individuals felt more confident in their ability to solve the tasks and also demonstrated better task performance. Additionally, task-specific SE and task performance were moderately positively associated (*r* = 0.27, *p* < .001), suggesting that individuals who have greater confidence in their ability to solve these tasks also demonstrate better task performance.

When considering sex and age, a small negative relationship was observed between task-specific SE and sex (*r* = −0.23, *p* = .001), indicating that males exhibited a greater confidence in their ability to solve the tasks compared to females. However, this confidence did not translate into actual task performance, as evidenced by the non-significant correlation between sex and task performance (*r* = −0.08, *p* = .250).

**Figure 2:**
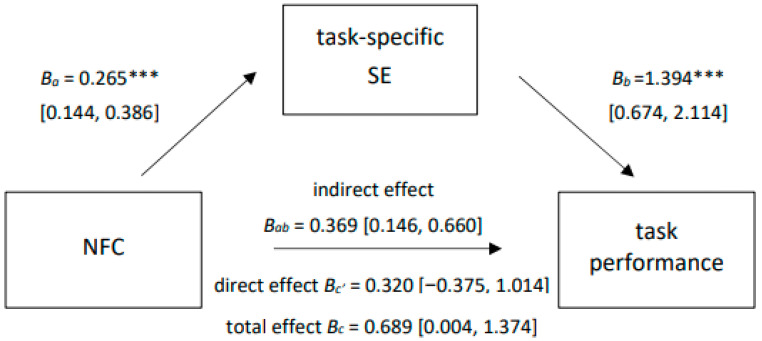
Mediation of Need for Cognition (NFC) on task performance through task-specific self-efficacy (SE), *N* = 204. All path coefficients are reported unstandardized. Values in squared brackets indicate upper and lower bound of a 95% confidence interval based on 5.000 percentile bootstraps; *** *p* < .001.

A simple mediation analysis (Process model 4, see Hayes 2017) was conducted to examine whether the effect of NFC on task performance would be mediated by the confidence in the ability to solve the tasks (Hypothesis 3). NFC was considered to be the independent variable, task performance as the dependent variable, and task-specific SE as the mediator. As shown in Figure 2, an effect of NFC on task performance was observed (*B*c = 0.689, *p* = .049). Moreover, NFC significantly predicted the mediator (*B*a = 0.265, *p* < .001) and the mediator in turn predicted task performance (*B*b = 1.394, *p* < .001), resulting in an indirect effect (*B*ab = 0.369, 95% CI [0.146, 0.660]). The model provides evidence for full mediation, as the direct effect of NFC on task performance was no longer significant when the mediator was included (*B*c’ = 0.320, *p* = .366). When sex, which was significantly associated with the mediator, was additionally included in the model as a covariate, the pattern of results remained nearly the same. Only the total effect of NFC on task performance slightly missed significance (*B*c = 0.653, *p* = .061).

In the next step, we extended the mediation model by incorporating the moderator variable “setting” to investigate whether the presence of the experimenter influenced the effect of NFC on task performance (research question). Specifically, we assessed whether the setting variable (alone vs. experimenter) moderates the direct path from NFC to task performance and whether it affects the indirect path through task-specific SE (Process model 15, see Hayes 2017). The setting variable did not have a significant effect on task performance, either directly (*B* = 0.947, *p* = .082) or in interactions with NFC (*B* = 0.696, *p* = .323) or task-specific SE (*B* = 0.871, *p* = .250). When examining the indirect effect of NFC on task performance via task-specific SE, the model produced a non-significant index of moderated mediation (Index = 0.231, 95% CI [−0.151, 0.689]), indicating the absence of moderated mediation and suggesting that the conditional indirect effects should not be interpreted. Therefore, the results imply that the presence of the experimenter during the task processing was largely irrelevant for both the direct and indirect effects of NFC on task performance. Additionally, including sex as a covariate in the model did not alter this pattern of effects.

## 4. Discussion

This paper aims to contribute to the understanding of the potential factors that mediate the relationship between NFC and task performance. Based on the literature suggesting that SE expectancies mediate the relationship between NFC and cognitive performance (Day et al. 2007; Elias and Loomis 2002; Naderi et al. 2018), we sought to investigate this relationship systematically under controlled laboratory conditions.

We examined the effects of NFC and SE on performance in a set of cognitively demanding tasks. The task set consisted of validated intelligence test tasks with challenging difficulty levels to create a context relevant for NFC-typical behavior. We hypothesized that individuals with higher NFC would perform better on reasoning tasks than those with lower NFC, that they would also be more confident in their abilities, and that this higher confidence would mediate the relationship between NFC and task performance. Additionally, we were interested in whether the visibility of the test subject’s performance to others (i.e., the experimenter) would influence these associations.

### 4.1. Relationship Between NFC and Task-Specific SE

First, we examined the relationship between NFC and task-specific SE to determine whether individuals with increasing NFC have more confidence in their ability to solve a range of cognitively demanding tasks. Following Cacioppo and Petty (1982), it seemed plausible that individuals, who are more inclined to engage in and enjoy effortful cognitive processing, gain confidence in their ability to successfully perform cognitively challenging tasks.

We found a moderate positive association between NFC and task-specific SE, indicating that individuals with higher NFC exhibit greater confidence in their ability to solve tasks. This finding aligns with previous results on the relationship between NFC and SE (e.g., Elias and Loomis 2002; Naderi et al. 2018; Pillai et al. 2011; Tan et al. 2020). While prior research has predominantly focused on general SE (Naderi et al. 2018; Pillai et al. 2011), which refers to an individual’s belief in their overall competence across various situations (e.g., Chen et al. 2004), or academic SE (Elias and Loomis 2002), defined as the belief in one’s capability to achieve academic goals (Bandura 1977), the current study specifically explored more specific forms of SE. Participants assessed their confidence to successfully complete a cognitive task battery. Thus, our study contributes to the existing literature by demonstrating that individuals with higher NFC approach real task situations with greater confidence than those with lower NFC.

### 4.2. Relationship Between NFC and Cognitive Task Performance

Secondly, we investigated whether individuals with higher NFC not only believe they are better able to solve tasks, but also whether they can actually do so—specifically, whether their beliefs are reflected in their performance. Indeed, we found a small positive association that reached statistical significance, indicating that individuals with higher NFC performed better on cognitively challenging tasks than those who reported lower NFC. These results align with previous findings regarding the relationship between NFC and psychometric intelligence—such as fluid intelligence (Fleischhauer et al. 2010; Hill et al. 2013), crystallized intelligence (Fleischhauer et al. 2010; Hill et al. 2013; Tidwell et al. 2000; Woo et al. 2007), as well as academic performance (Grass et al. 2017; Strobel et al. 2024). Over the past few decades, several theoretical frameworks have been proposed to explain how cognitive motivation and cognitive performance might be related. For example, Hill et al. (2013) suggested that NFC might influence certain aspects of intelligence reflected in specific cognitive performance during development as a result of a greater persistence in cognitive challenges and exposure to information making them more able to successfully work on such demanding tasks. Conversely, Ackerman (1996) proposed in his “intelligence-as-process, personality, interests, and intelligence-as knowledge” model (PPIK) that associations between intelligence and traits reflecting cognitive motivation may arise because success in an intelligence task leads intellectually capable individuals to develop a greater interest in engaging with such problems more frequently. A lack of success, reflecting lower abilities, is posited to have detrimental effects on an individual’s interests and the development of personality traits associated with cognitive motivation (Ackerman 1996). Both assumptions are integrated into the OFCI model by Ziegler et al. (2012), which is a developmental process model linking the NFC-related factor of openness to experiences with fluid and crystallized intelligence. It is argued that openness leads people into richer environments that helps them practice and thus positively influence the development of their fluid intelligence (environmental enrichment hypothesis). Similarly, the reverse effect is also conceivable: individuals with higher fluid intelligence are more likely to succeed in challenging new tasks, which can lead to a positive perception of these situations and an increased enjoyment of thinking and problem-solving (environmental success hypothesis). In addition, a joint influence of openness and fluid intelligence on crystallized intelligences is assumed in the OFCI model.

### 4.3. Task-Specific SE as Mediator of the Relationship Between NFC and Cognitive Task Performance

It is important to note that these theoretic ideas aim to explain the association between NFC and cognitive task performance from a developmental perspective, without directly addressing the factors through which NFC may be associated with cognitive performance in a very specific testing situation. This was the focus of our third hypothesis. Here, we investigated whether the confidence of individuals with high NFC influences their ability to solve the tasks. The observed indirect effect in the mediation model, however, supports the assumption that the associations between NFC and cognitive performance are also due to NFC’s positive influence on task-specific SE, which in turn may impact on cognitive performance. This expands upon previously identified differences in how individuals with higher and lower NFC approach cognitive challenges. For instance, some findings suggest that those with higher NFC do not necessarily complete more tasks than those with lower NFC, but they are more correct in the tasks they try to solve, suggesting a greater cognitive effort in solving a task (Fleischhauer et al. 2010). The present research, which identifies task-specific SE, adds another potential situational factor to this discussion.

Although we have introduced the four variables measuring a participant’s confidence in solving a respective type of task as measures of task-specific SE towards these, there is of course reason to suspect that a more general SE towards cognitive challenges is predictive of these task-specific beliefs. However, the data on these variables was collected just after participants saw the sample tasks and the items were asking for their confidence towards just these specific tasks (and not, e.g., confidence in generally solving spatial intelligence tasks or inductive reasoning tasks). We therefore do not think that what we have here labeled “task-specific SE” is merely an operationalization of general SE towards cognitive challenges, but instead is specific for the tasks we used. We thank an anonymous reviewer for suggesting and clarifying that.

### 4.4. Role of Performance Visibility in the Interplay Between NFC, Task-Specific SE, and Cognitive Task Performance

Lastly, we examined the role of performance visibility in the interplay between NFC, task-specific SE, and task performance. Participants solved the task in the laboratory without the presence of an experimenter (setting 1), or with the experimenter in the room observing the performance of the participants (setting 2). Various studies indicate that the pressure to perform in front of others can induce nervousness or anxiety, which in turn may impair performance, particularly when the task is perceived as challenging or novel (e.g., Rezaei et al. 2017). However, individuals with higher NFC report greater enjoyment in engaging with cognitively demanding tasks (Amabile et al. 1994) and are likely to perceive these tasks as less stressful. Additionally, as shown by the study of Steinhart and Wyer (2009), they are more motivated to avoid failure when confronted with a challenging task. Building on these findings, we hypothesized that the perceived stress due to the presence of others might be less pronounced for individuals with higher NFC. Accordingly, we presumed that the positive relationship between NFC and task performance, along with the indirect effect through task-specific SE, could be more pronounced in setting 2 than in setting 1. However, the results indicate that the presence of an experimenter was largely irrelevant for both the direct and indirect effects of NFC on task performance. One possible explanation for this could be that individuals with high NFC are motivated to avoid failure from an intrinsic drive rather than due to the presence of others. Given that NFC negatively correlates with concerns about social demands (Osberg 1987) and social identity orientation (Cacioppo et al. 1986), it appears to be a stronger motivation for individuals with higher NFC to avoid an incompetent self-image rather than to showcase their abilities (Steinhart and Wyer 2009).

Additionally, our sample showed a negative correlation between sex and task-specific SE, with men being more likely than women to believe they could successfully solve the cognitively demanding tasks, despite no significant difference in actual performance as indicated by the insignificant correlation between sex and task performance. When sex was included as a covariate in the mediation model, the indirect effect of NFC on task performance via SE remained significant, indicating that sex did not serve as an underlying factor in the interplay between NFC and SE on task performance. The frequent observation that women rate their cognitive ability lower than men (e.g., Furnham 2017) is often attributed to the “male hubris, female humility” effect (Furnham et al. 2001). One potential explanation for this phenomenon, particularly in relation to math and logical thinking, could be the persistence of gender stereotypes (e.g., Ertl et al. 2017).

### 4.5. Limitations and Future Directions

Several limitations warrant discussion and should be addressed in future research. One limitation of this study is the relatively homogeneous sample, which predominantly consisted of psychology students. This group not only has familiarity with psychological questionnaires and testing procedures, but may also exhibit a more restricted variance in the measured variables compared to a representative sample. The potential impact on the results could be mitigated by the fact that the sample primarily comprised students with limited experience in testing procedures, as opposed to university graduates. Nonetheless, due to this restricted variance, it is likely that the correlations found in the present study are underestimated rather than overestimated.

Furthermore, it should be noted that the cross-sectional design of the present study may limit the interpretation of the mediation analyses. However, since NFC (independent variable) was measured at an earlier time point than task performance (dependent variable), and since the task-specific SE (mediator) refers to a specific task that was previously demonstrated, it is much more likely that NFC influences task performance through the mediator variable rather than the mediator variable exerting influence on task performance through NFC. Support for this interpretation is also given by Elias and Loomis (2002), who have tested two competing mediation models against each other with NFC and academic SE, respectively, serving as mediators. In their study, only academic SE served as mediator of the relationship between NFC and academic performance, but not vice versa.

We chose a task battery with items of higher difficulty to create a cognitively demanding situation. However, this higher level of difficulty was also associated with limited response variance, as only about 10% of the items were solved on average. It is therefore likely that the correlations of NFC and SE with task performance were somewhat underestimated. Since no normative data were available for the task battery, the standard deviation of the population is unknown, making it difficult to correct for range restriction. Moreover, the range restriction in our case is not due to a selective choice of test subjects; rather, it reflects floor effects resulting from the difficulty of the tasks. Even in a larger and heterogeneous sample, the average of correctly answered tasks, as well as the variance, would likely also be low. Another possible explanation for the low variance in task performance could be the response option “I don’t know the answer.” It cannot be ruled out that participants opted for this answer to avoid making an incorrect response, rather than attempting to answer each task to the best of their ability.

Our study has shown that the relationship between NFC and cognitive performance is also due to the positive influence of NFC on task-specific SE, which in turn affects cognitive performance. An important question for future research concerns the potential mediators that could explain the relationship between these specific beliefs about one’s confidence in solving cognitive challenges and actual performance. For instance, individuals who approach such tasks with greater confidence may be better protected against the negative effects of minor setbacks such as failing to solve a task. Such individuals might also participate in a testing situation with a less negative or even anxious mood, which could potentially reduce the adverse effects of these emotions on task performance. While investigating these potential mediators could significantly enhance our understanding of how beliefs and actual performance are interconnected, measuring the relevant variables and demonstrating causal relationships remains a challenge.

In this study, we build on previous research by examining the role of performance visibility (i.e., whether task performance observed by the experimenter or not) in the interplay between NFC, task-specific SE, and cognitive task performance. The non-significant effects suggest that performance visibility may not be a relevant moderator for the interaction of NFC and task-specific SE on cognitive performance. Future research should explore additional potential moderators. For instance, Niemiec and Lachowicz-Tabaczek (2015) investigated mood states as a potential moderator in the relationship between task-specific SE and cognitive performance and showed that this relationship was only existent during a positive mood.

## Figures and Tables

**Table 1:** Descriptives and intercorrelations of variables of interest.

	*M*	*SD*	1	2	3	4	5
1. NFC	0.79	0.81	**0.85**	0.29 ***	0.14 *	−0.11	0.10
2. task-specific SE	2.59	0.73		**0.77**	0.27 ***	−0.23 **	−0.04
3. task performance	9.59	4.03			**0.60**	−0.08	−0.02
4. sex (0 = male, 1 = female)	-	-				**-**	−0.20 **
5. age	23.12	3.67					**-**

Notes. *N* = 204, * *p* < .05, ** *p* < .01, *** *p* < .001; Cronbach’s α indicators are displayed in bold on the diagonal. NFC = Need for Cognition, SE = self-efficacy.

## Data Availability

The dataset generated and analyzed in the current study is available at: https://osf.io/cjq46/ (accessed on 25 November 2024).
